# Popliteal Aneurism; Tying of the Femoral Artery; Recovery

**Published:** 1850-11

**Authors:** 


### SURGERY.

*Popliteal Aneurism; Tying of the Femoral Artery; Recovery. A New Aneurism-Needle.*—It is interesting to look back on the various phases of the treatment of external aneurism, and modern surgery may well be proud of the gigantic strides which have been made within the last hundred years. Hunter's and Scarpa's share in these improvements must ever give them a place (where this even their only merit) among the benefactors of the human race. But great credit is likewise due to surgeons of our own times for reviving the use of compression, regulated so as to lessen the force of the circulation, and to promote the solidification of the aneurismal tumour; and the Irish school has done much to promote the improvements in the instruments used for that purpose. Dr. Bellingham, who has worthily labored in the cause of compression, has not yet succeeded in converting all his professional brethren to his opinion, if we are to judge from the London practice, and from the condemnations which have issued from North Britain. As the question is still *sub judice*, it would be rash and presumptuous to offer a decided opinion on the subject; but we will venture to say, that it would be extremely useful if a competent surgeon would take the trouble of partially examining the question, and give the profession his opinion, not whether one of the two lines of practice (the ligature or compression) is the best, but in which cases and under what circumstances, either is to be applied.

It will be perceived by the following case, that Mr. Le Gros Clark is not wedded to any particular mode of treating such cases, for he would have tried compression whilst his patient was in a favourable condition for such treatment, but resorted to the ligature when delay had put other means out of the question. We proceed to give an outline of the case.

The patient is a man of forty-eight, robust, and of florid complexion, accustomed to active exercise, being a woodman on a large estate. He was admitted into St. Thomas's Hospital, June 15th, under the care of Mr. Le Gros Clark. About six months before the patient presented himself at the hospital, his attention was attracted to the affected limb by a pain extending from the hip to the ankle, and especially about the knee. He applied to Mr. Curtis, of Harting, Sussex, who at once detected a swelling in the ham, and ascertained its nature to be aneurismal. There was some œdema of the leg at the time. The patient was first seen by Mr. Clark, March 24th, who urged him to enter the hospital at once, but the patient declined doing so. The tumor was *then* about the size of a duck's egg, and occupied the upper and *outer* part of the popliteal space; it pulsated strongly, but the impulse could be arrested by pressure on the femoral artery, and the sac partially emptied. When admitted in June, however, nearly the *whole* ham was occupied by the tumour, which was also much more solid than before, from the increased deposit of fibrine, so that but little impression could be made on the sac by pressure on the artery. The pain in the limb was very great, and described as sometimes excessive; the thigh and leg presenting a certain amount of œdema and venous congestion. The patient had been following his usual occupations up to this admission, his health was good, and the viscera of the chest were found in a normal state.

After the man had been duly prepared, Mr. Le Gros Clark tied the femoral artery, in the usual way, five days after admission. The patient proved very excitable both during and after the operation. In the evening the temperature of the operated limb was slightly below that of the sound one; there was no pulsation either in the sac or arteries; and as the excitability continued, twenty minims of Battley's solution were ordered, and to be repeated if necessary. Two doses were required to procure rest, and on the next day the wound looked healthy; the pulse was 106, the skin hot and thirst great. Temperature of the popliteal space on the operated side, 101°, on the sound side 99½°. On the second day, Mr. Clark, finding his patient rather weak, ordered two ounces of gin, which had been the man's accustomed beverage; and on the third he expressed a desire for food. The wound looking rather sluggish, the diet was gradually increased, and the patient improved rapidly. On the fifteenth day the ligature came away in the dressings, and the patient returned home (the wound being nearly healed) about one month after the operation; there had been no pulsation either in the sac or arteries, below the ligature, since the latter had been applied. The patient was seen about three weeks after his discharge, and was doing well.

Mr. Le Gros Clark remarked at the time of the operation, that when he saw the patient at an earlier period he urged him to submit at once to treatment for the cure of the disease; and he had intended trying compression on the femoral artery at two different points, so as to keep up a certain amount of resistance to the flow of blood without risk to the superjacent soft parts. The progress of the disease, the increased size of the tumor, now occupying the entire popliteal space; the œdema of the limb, the suffering of the patient, and his extreme constitutional excitability all concurred in forbidding that course. The application of a ligature on the trunk of the artery, in the usual position, was, therefore the only alternative which offered itself.

Mr. Clark used in this operation a new aneurism needle, manufactured by Mr. Bigg, of St. Thomas's-street.

This needle is composed of an oval silver stem, a portion of the the extremity of which (about half an inch) is hollow, to receive the point, which is bulbous, and takes off at will. This bulb has a thin prolongation backwards, containing the eye, so that the ligature is concealed within the cavity of the bend of the instrument, and hangs loosely from its posterior aspect, a few lines below the beginning of the curve. The thread being thus disposed, offers no obstruction whilst the needle is passing under the artery; and when the operator finds it necessary to cut through any fascia that may resist the point of the instrument, he does not run the risk of dividing the silk. The needle is furnished with an ivory handle, having two deep notches cut in the end, for the purpose of winding the ligature round the extremity of the handle, and rendering the instrument firm. Simplicity seems to be the great quality of this needle; for in Mott's the point is screwed off from behind the curve; in Kirby's there is a loose pair of forceps resting in a groove, which seizes the point of the needle, and withdraws it from the sheath or handle; and Le Strange has added a sliding canula to Mott's needle by which the screw is fixed, and the possibility of the point shifting during the operation prevented. In fact, several aneurism needles have been invented to accomplish the purposes above described, but, from their complicated nature, have one after another been laid aside,—*London Lancet.*

